# The effects of *SCARB2* and *SELPLG* gene polymorphisms on EV71 infection in hand, foot, and mouth disease

**DOI:** 10.17305/bb.2023.8948

**Published:** 2023-10-01

**Authors:** Fengyuan Duan, Zengqing Du, Yang Wang, Lan Luo, Lijiang Du, Hong Jiang, Yantuanjin Ma, Yuling Yang

**Affiliations:** 1 Department of Cell Biology and Medical Genetics, Kunming Medical University, Kunming, China; 2 Kunming Kingmed Institute for Clinical Laboratory Co., Ltd., Kunming, China; 3 Infectious Disease Department, Kunming Children’s Hospital, Kunming, China; 4 Clinical Laboratory, Yan’an Hospital of Kunming City, Kunming, China

**Keywords:** Hand-foot-and-mouth disease (HFMD), genetic polymorphism, enterovirus 71 (EV71), selectin P ligand (*SELPLG*), scavenger receptor class B member 2 (*SCARB*2)

## Abstract

The same viral infection in different hosts may result in varying levels of clinical symptoms, which is related to the genetic background of the host itself. A total of 406 common cases and 452 severe cases of enterovirus 71 (EV71) infection in Yunnan Province were selected as the research subjects, and SNaPshot technology was used to detect genetic polymorphisms for 25 Tag single-nucleotide polymorphisms (TagSNPs) in the selectin P ligand (*SELPLG*) and scavenger receptor class B member 2 (*SCARB2*) genes. Our results demonstrate that *SCARB2* polymorphisms (rs74719289, rs3733255, and rs17001551) are related to the severity of EV71 infection (A vs G: odds ratio [OR] 0.330; 95% confidence interval [CI] 0.115–0.947; T vs C: OR 0.336; 95% CI 0.118–0.958; and A vs G: OR 0.378; 95% CI 0.145–0.984). The *SELPLG* polymorphisms were not significantly different between common cases and severe cases. Therefore, we conclude that the *SCARB2* gene has a protective effect on the course of hand, foot, and mouth disease caused by EV71 infection and that *SCARB2* gene mutations can reduce the severity of the disease.

## Introduction

Hand-foot-and-mouth disease (HFMD) is an infectious disease caused by a variety of enteroviruses, mainly spread through the fecal–oral route and inhalable respiratory droplets [1]. The patients are usually less than five years old. Most patients have symptoms, such as fever, recurrent aphthous ulcer, and skin rashes on the hands, feet, and buttocks [2]. A few patients have experienced encephalitis, flaccid paralysis, and even death. The HFMD epidemic has broken out in the Asia–Pacific region, posing a serious public health threat. Furthermore, the pathogenic mechanism of HFMD and the biological characteristics of the virus have not been fully elucidated.

HFMD is mainly caused by enterovirus A71 (EV71) [3, 4], and EV71 infection might cause neurological, psychiatric complications, and even death [5]. In clinical practice, the symptoms of HFMD patients are usually mild and self-limiting, but a severe EV71 infection can lead to a diverse array of neurological diseases. Therefore, the same viral infection in different hosts may result in variations in clinical symptoms, which is not only related to the virulence of EV71 but also dependent on the immune responses of different hosts.

EV71 infection is affected by cell surface receptors, including the human scavenger receptor class B member 2 (SCARB2), and attachment receptors, such are P-selectin glycoprotein ligand-1 (PSGL-1). SCARB2 is encoded by the *SCARB2* gene, and it was mainly observed in lung pneumocytes, hepatocytes, renal tubular epithelium, splenic germinal centers, intestinal epithelium, and most central nervous system (CNS) neurons [6, 7]. It can shuttle between endosomes, lysosomes, and plasma membranes using membrane flow [8]. SCARB2 plays a crucial role in EV71 infection by mediating viral attachment, internalization, and uncoating through the clathrin-mediated endocytic pathway [9, 10]. Attachment receptors are thought to support EV71 attachment to the cell surface and enhance EV71 infection by increasing a probability of encountering a true receptor. These molecules include PSGL-1, heparan sulfate, annexin II, sialic acid, nucleolin, and vimentin [11]. PSGL-1 is encoded by the *SELPLG* gene. As an adhesion molecule involved in immune cell trafficking, it is recognized as a regulator of immune responses [12]. EV71 strains are classified into two distinct phenotypes according to PSGL-1-binding capability: PSGL-1-binding (PB) and PSGL-1-nonbinding (non-PB) strains [13]. Studies in cynomolgus monkeys showed that non-PB strains were more virulent than PB strains [11], However, in some molecular epidemiologic studies, VP1-145G/Q viruses (PB strains) were isolated more frequently from severely affected patients than from mildly affected patients [14–17], which seems to indicate that the PB strains are more virulent in humans. These apparently contradictory findings in humans and animal models are yet to be studied.

Several gene polymorphisms in cytokines and chemokines, such as interferon gamma (IFN-γ), interleukin 8 (IL-8), interleukin 10 (IL-10), interleukin 17F (IL-17F), C–C motif chemokine ligand 2, and C–X–C motif chemokine 10, have been reported to be associated with susceptibility to EV71 infection [18]. This suggests that host genetic factors can play an important role in EV71 infection. The different genetic polymorphisms of *SELPLG* and *SCARB2* in different individuals may lead to differences in the expression of PSGL-1 and SCARB2 proteins, which may directly affect the efficiency of virus entry into cells and the subsequent emergence and strength of cellular immune responses, ultimately leading to differences in the degree of patient infection.

In this study, a case-control association study was performed and HFMD patients infected with the same virus strain (C4 EV71) were selected as the study subjects to exclude the impact of the different virus strains. We investigated the effects of *SELPLG* and *SCARB2* gene polymorphisms in EV71 infection and looked for susceptibility to EV71 infection. This study could provide a valuable research basis for exploring the pathogenic mechanism of HFMD and factors affecting the severity of the disease.

## Materials and methods

### Cases and diagnostic criteria

**Figure 1.**
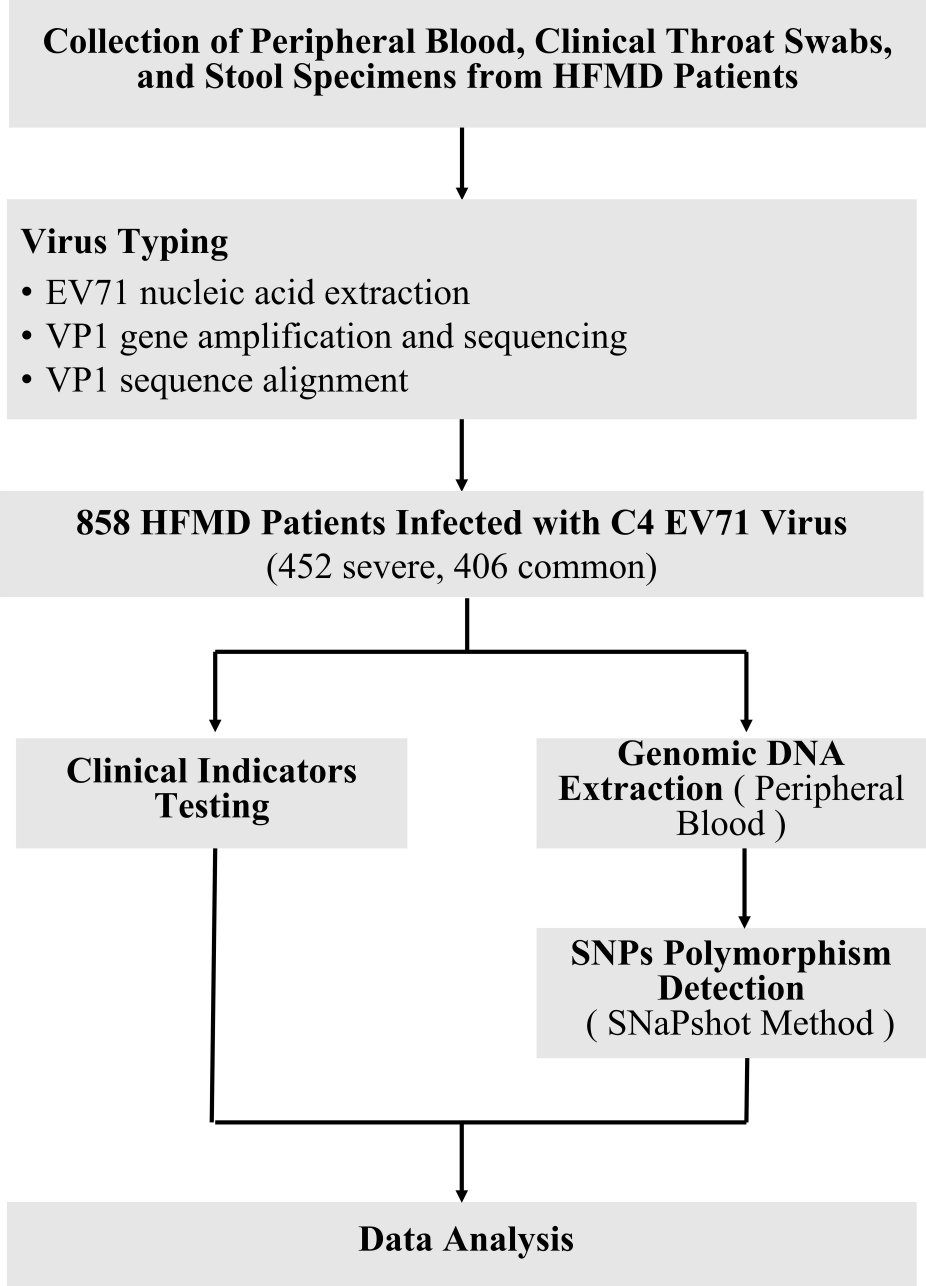
**The flowchart of the study.** HFMD: Hand, foot, and mouth disease; EV71: Enterovirus 71; SNP: Single-nucleotide polymorphism.

In this study, HFMD patients infected with C4 EV71 virus who were admitted to hospital between 2017 and 2021 were the research subjects, including 452 severe cases (276 males and 176 females) and 406 common cases (245 males and 161 females). Diagnostic criteria for HFMD were determined according to the Guidelines for the Diagnosis and Treatment of Hand Foot and Mouth Disease (2018 version), issued by the National Health Commission of the People’s Republic of China and the Textbook of Pediatrics. Common cases involved patients who had skin rashes on the hands, feet, mouth, and buttocks, which may be accompanied by cough, runny nose, loss of appetite, etc. Severe cases included patients who had CNS involvement, listlessness, lethargy, weak sucking, hyperarousal, headache, vomiting, fidgeting, limb shaking, myasthenia, stiff neck, etc. Critical cases included patients who demonstrated shortness of breath, cyanosis of the lips, pink foamy sputum or bloody fluid, decreased blood pressure, or shock. Children with HFMD who were admitted to the hospital for more than ten days or were admitted to the hospital due to other diseases during the recovery period were excluded from the study. The flowchart of the study is shown in Figure 1.

### Sample collection and pathogenesis testing

For clinical throat swabs collection, the patient opened the mouth, and sample collector wiped their tonsils and posterior pharyngeal wall back and forth with a disposable sterile sampling swabs three times, and then placed the swab into the sampling tube. For stool sample collection, approximately 3–5 g of patient stool was collected and placed in a sterile container. Nucleic acid was extracted from clinical throat swabs or stool samples of suspected cases using an EV71 nucleic acid detection kit (Jiangsu Mole Bioscience Co., Ltd.) according to the manufacturer’s protocol. EV71 nucleic acid positive samples were selected, PCR amplification of the *VP1* gene was performed as described by Wang et al. [19]. After purification, *VP1* gene amplification products were sequenced by Sanger sequencing technology using an ABI3730XL automatic DNA sequence analyzer (Applied Biosystems, USA). DNAStar MegAlign software was used to compare the homology of the sequencing results with the EV71 virus *VP1* gene sequence in GenBank to confirm C4 EV71 virus infection.

### Determination of clinical indicators

Venous blood was collected from infected subjects. An automatic hematology analyzer was used to test blood indicators, including hematocrit (HCT), hemoglobin (HGB), absolute value of lymphocyte (LYMPH), mean corpuscular hemoglobin (MCH), MCH concentration (MCHC), mean corpuscular volume (MCV), absolute value of monocytes (MO), absolute value of neutrophils (NEUT), platelets (PLT), red blood cells (RBC), red blood cell volume distribution width (RDW), and white blood cells (WBC).

### Tag single-nucleotide polymorphisms (TagSNPs) selection and analysis

Required data were downloaded from 1000 Genomes Browser (https://www.ncbi.nlm.nih.gov/variation/tools/1000genomes/). TagSNPs were selected using HaploView4.2 software (the upstream and downstream settings range was 2K, MAF ≥ 0.05, *R*^2^ ≥ 0.8). Eight TagSNPs for the *SELPLG* gene and 17 TagSNPs for the *SCARB2* gene were obtained (Table S1). The SNaPshot method was used to analyze the polymorphisms of the single-nucleotide polymorphism (SNP) sites. Primers used in our study are shown in Table S2.

### Ethical statement

The study protocol was conducted following the Declaration of Helsinki and approved by the Medical Ethics Committee of Kunming Medical University (KMMU2021MEC055). Informed and written consent was obtained from the parents/legal guardians of all subjects involved in the study.

### Statistical analysis

The Hardy–Weinberg equilibrium test and the chi-square test for the genotype frequency and allele frequency were performed using SHEsis software [20]. Genetic model analysis for all SNPs was performed using PLINK software. Logistic regression analysis was performed using SPSS23.0 for each SNPs’ genotype and allele to derive their correlation with the severity of EV71 infection. Continuous variables were represented as mean ± SD. The t-test was used for comparison between groups for measurement data, and the chi-square test was used for comparison between groups for counted data. *P* < 0.05 indicated statistical significance.

## Results

### Clinical and biochemical indicators

There was no significant difference in sex or age between the severe case group and the common case group of HFMD. In the severe group, the NEUT was higher than in the common case group, and the RBC and HGB levels were lower than those in the common case group (*P* < 0.05). The LYMPH and PLT were higher than their respective reference values, and the MCV and MCH were lower than their respective reference values for both groups. The other indicators were within the reference value ranges (Table 1).

**Table 1:** Clinical and biochemical indicators of the common case group and the severe case group

**Parameters**	**Reference values**	**Common case group (*n* ═ 406)**	**Severe case group (*n* ═ 452)**	**t-test*/X^2^***	***P* value**
Sex (M/F)	/	245 / 161	276 / 176	0.046	0.830
Age (years)	/	3.350 ± 1.927	3.220 ± 1.626	0.113	0.737
WBC (×10^−9^/L)	3.5 ∼ 9.5	8.331 ± 3.051	8.957 ± 2.733	0.935	0.333
NEUT (×10^−9^/L)^Δ^	1.8 ∼ 6.3	3.582 ± 2.074	5.030 ± 2.518	6.159	**0.014**
LYMPH (×10^−9^/L)^*^	1.1 ∼ 3.2	4.297 ± 1.949	3.418 ± 1.866	3.381	0.066
MO (×10^−9^/L)	0.1 ∼ 0.6	0.730 ± 0.514	0.611 ± 0.853	0.366	0.545
RBC (×10^−12^/L)^Δ^	3.8 ∼ 5.1	4.941 ± 0.332	4.686 ± 0.370	8.033	**0.005**
HGB (g/L)^Δ^	115 ∼ 150	131.200 ± 10.670	125.165 ± 10.929	5.392	**0.020**
HCT (%)	40 ∼ 50	38.855 ± 2.891	36.799 ± 4.299	3.853	0.050
MCV (fL)^*^	82.0 ∼ 100.0	79.640 ± 4.214	79.433 ± 6.380	0.020	0.886
MCH (pg)^*^	27.0 ∼ 34.0	26.730 ± 1.780	26.954 ± 2.008	0.231	0.631
MCHC (g/L)	316.0 ∼ 354.0	335.500 ± 10.995	336.500 ± 23.992	0.034	0.854
RDW (fL)	41.2 ∼ 53.6	39.155 ± 2.319	38.994 ± 2.908	0.059	0.809
PLT (×10^−9^/L)^*^	125 ∼ 350	324.550 ± 90.371	345.025 ± 86.787	1.006	0.316

*Deviation from the reference value. ^Δ^There was a significant difference between the common case group and the severe case group, *P* < 0.05. Bold values indicate statistical significance. *X^2^*: Chi-square test; WBC: White blood count; NEUT: Absolute value of neutrophils; LYMPH: Absolute value of lymphocytes; MO: Absolute value of monocytes; RBC: Red blood cells; HGB: Hemoglobin; HCT: Hematocrit; MCV: Mean corpuscular volume; MCH: Mean corpuscular hemoglobin; MCHC: Mean corpuscular hemoglobin concentration; RDW: Red blood cell volume distribution width; PLT: Platelets.

### Allele frequency in the common case group and the severe case group

Among total 25 TagSNPs, the allele frequencies of rs74719289 A (odds ratio [OR] 0.330; 95% confidence interval [CI] 0.115–0.947; *P* ═ 0.031), rs3733255 T (OR 0.336; 95% CI 0.118–0.958; *P* ═ 0.033), rs17001551 A (OR 0.378; 95% CI 0.145–0.984; *P* ═ 0.039), and rs894250 C (OR 0.378; 95% CI 0.145–0.984; *P* ═ 0.039) in the severe case group were lower than those of the common case group for the *SCARB2* gene (*P* < 0.05). No significant differences in allele frequency for the remaining SNPs were detected between the common case group and the severe case group (Table 2).

**Table 2:** Distributions of allele frequencies in the common case group and the severe case group

**Gene**	**SNPs**	**Allele**	**Minor allele**	**Minor allele common cases (*n*, %)**		**Minor allele severe cases (*n*, %)**		**OR (95% CI)**	***X^2^***	***P* value**
*SCARB2*	**rs17001551**	A/G	A	122	15.02	56	6.19	0.378 (0.145–0.984)	4.251	**0.039**
*SCARB2*	rs35583533	C/T	C	162	19.95	204	22.68	1.178 (0.524–2.646)	0.158	0.691
*SCARB2*	rs3733256	C/G	C	108	13.15	50	5.45	0.384 (0.136–1.084)	3.493	0.062
*SCARB2*	rs6825004	G/C	G	204	25.00	283	31.22	1.358 (0.644–2.864)	0.649	0.421
*SCARB2*	rs8475	A/T	A	325	40.15	357	39.60	0.977 (0.502–1.898)	0.005	0.944
*SCARB2*	rs894251	A/G	A	406	50.12	400	44.25	0.794 (0.414–1.521)	0.486	0.486
*SCARB2*	**rs74719289**	A/G	A	102	12.56	41	4.46	0.330 (0.115–0.947)	4.646	**0.031**
*SCARB2*	rs76229059	G/A	G	235	28.91	300	33.15	1.227 (0.590–2.552)	0.302	0.583
*SCARB2*	rs1051326	C/G	C	345	42.49	358	39.64	0.885 (0.458–1.710)	0.133	0.716
*SCARB2*	rs3796498	T/C	T	162	19.95	135	14.93	0.706 (0.310–1.606)	0.696	0.404
*SCARB2*	rs9991821	A/G	A	122	15.02	167	18.44	1.267 (0.513–3.131)	0.264	0.607
*SCARB2*	rs17001640	G/A	G	325	40.15	392	43.36	1.143 (0.589–2.218)	0.157	0.692
*SCARB2*	rs6824953	C/G	C	203	25.00	284	31.36	1.366 (0.647–2.882)	0.673	0.412
*SCARB2*	**rs894250**	C/A	C	122	15.02	56	6.19	0.378 (0.145–0.984)	4.251	**0.039**
*SCARB2*	**rs3733255**	T/C	T	107	13.15	43	4.79	0.336 (0.118–0.958)	4.54	**0.033**
*SCARB2*	rs11547135	C/T	C	385	52.61	396	43.69	0.693 (0.356–1.350)	1.173	0.279
*SCARB2*	rs1465922	A/G	A	235	28.91	331	36.62	1.396 (0.673–2.897)	0.807	0.369
*SELPLG*	rs2228315	T/C	T	284	35.10	287	31.75	0.854 (0.431–1.691)	0.205	0.65
*SELPLG*	rs3782522	T/C	T	406	50.00	364	38.27	0.626 (0.326–1.201)	2.012	0.156
*SELPLG*	rs765267	G/A	G	204	25.00	225	24.89	0.987 (0.466–2.091)	0.001	0.972
*SELPLG*	rs8179133	A/G	A	203	24.88	230	25.22	1.013 (0.479–2.146)	0.001	0.972
*SELPLG*	rs4964269	A/G	A	386	47.54	405	44.69	0.895 (0.467–1.717)	0.111	0.739
*SELPLG*	rs7138370	G/C	G	204	24.88	191	23.28	0.921 (0.434–1.954)	0.046	0.83
*SELPLG*	rs1981758	T/C	T	223	27.46	240	26.55	0.963 (0.465–1.995)	0.01	0.919
*SELPLG*	rs8179141	T/C	T	142	17.49	125	13.75	0.744 (0.314–1.767)	0.451	0.502

Bold values indicate statistical significance. SNPs: Single-nucleotide polymorphisms; OR: Odds ratio; CI: Confidence interval; *X^2^*: Chi-square test.

### Genotype frequency in the common case group and the severe case group and genetic model analysis

For rs17001551, the alleles are A and G. The A allele is the minor allele. The genotype frequencies of the GG, GA, and AA in the severe case group were 87.56%, 12.44%, and 0.00%, respectively, while they were 70.00%, 30.00%, and 0.00% (*P* ═ 0.031) in the common case group, respectively (Table 3). The A allele has a lower frequency in the population and is considered as a mutant gene. Patients carrying this mutation experienced milder symptoms in a dominant model (AA + GA vs GG, OR 0.331; 95% CI 0.117–0.942; *P* ═ 0.038). This difference was not statistically significant in a recessive model (AA vs GA + GG). Therefore, we suggest that if the A allele is associated with the severity of EV71 infection, it might play a role in a dominant model (Table 4).

**Table 3:** Distributions of genotype frequencies in the common case group and the severe case group

**Gene**	**SNPs**	**Common case group**							**Severe case group**							***P* value**
		**MM (n, %)**		**MN (n, %)**		**NN (n, %)**		**Total**	**MM (n, %)**		**MN (n, %)**		**NN (n, %)**		**Total**
*SCARB2*	**rs17001551**	284	70.00	122	30.00	0	0.00	406	396	87.56	56	12.44	0	0.00	452	**0.031**
*SCARB2*	rs35583533	244	60.00	162	40.00	0	0.00	406	263	58.21	173	38.31	16	3.48	452	0.698
*SCARB2*	rs3733256	297	73.68	106	26.32	0	0.00	403	392	89.06	48	10.94	0	0.00	440	0.052
*SCARB2*	rs6825004	203	50.00	203	50.00	0	0.00	406	216	48.00	187	41.50	47	10.50	450	0.297
*SCARB2*	rs8475	122	30.00	244	60.00	41	10.00	406	177	39.49	188	42.05	83	18.46	447	0.287
*SCARB2*	rs894251	102	25.00	203	50.00	102	25.00	406	151	33.33	202	44.78	99	21.89	452	0.750
*SCARB2*	**rs74719289**	305	75.00	102	25.00	0	0.00	406	398	91.01	39	8.99	0	0.00	437	**0.027**
*SCARB2*	rs76229059	191	47.37	191	47.37	21	5.26	403	199	44.39	201	44.90	48	10.71	448	0.755
*SCARB2*	rs1051326	122	30.00	223	55.00	61	15.00	406	180	40.10	182	40.61	87	19.29	449	0.462
*SCARB2*	rs3796498	264	65.00	122	30.00	20	5.00	406	333	73.63	103	22.89	16	3.48	452	0.707
*SCARB2*	rs9991821	284	70.00	122	30.00	0	0.00	406	300	66.67	134	29.80	16	3.54	450	0.692
*SCARB2*	rs17001640	122	30.00	244	60.00	41	10.00	406	148	32.84	216	47.76	88	19.40	452	0.480
*SCARB2*	rs6824953	203	50.00	203	50.00	0	0.00	406	215	47.96	185	41.33	48	10.71	448	0.289
*SCARB2*	rs894250	284	70.00	122	30.00	0	0.00	406	398	88.06	52	11.44	2	0.50	452	0.062
*SCARB2*	**rs3733255**	297	73.68	106	26.32	0	0.00	403	406	90.36	43	9.64	0	0.00	449	**0.027**
*SCARB2*	rs11547135	85	21.05	212	52.63	106	26.32	403	178	39.30	153	33.83	121	26.87	452	0.195
*SCARB2*	rs1465922	170	42.11	233	57.89	0	0.00	403	202	44.78	169	37.31	81	17.91	452	0.070
*SELPLG*	rs2228315	162	40.00	203	50.00	41	10.00	406	211	46.77	193	42.79	47	10.45	452	0.818
*SELPLG*	rs3782522	61	15.00	284	70.00	61	15.00	406	180	39.80	198	43.78	74	16.42	452	0.058
*SELPLG*	rs765267	203	50.00	203	50.00	0	0.00	406	252	55.72	175	38.81	25	5.47	452	0.412
*SELPLG*	rs8179133	223	55.00	162	40.00	20	5.00	406	254	56.22	166	36.82	31	6.97	452	0.924
*SELPLG*	rs4964269	81	20.00	264	65.00	61	15.00	406	151	33.33	198	43.78	103	22.89	452	0.191
*SELPLG*	rs7138370	223	55.00	162	40.00	20	5.00	406	267	59.30	156	34.67	27	6.03	451	0.889
*SELPLG*	rs1981758	203	50.00	183	45.00	20	5.00	406	250	55.22	164	36.32	38	8.46	452	0.695
*SELPLG*	rs8179141	264	65.00	142	35.00	0	0.00	406	338	74.87	102	22.61	11	2.51	451	0.384

Bold values indicate the single nucleotide polymorphisms that differ between the two groups. SNP: Single-nucleotide polymorphisms; M: Major allele; N: Minor allele.

**Table 4:** Statistical analysis of dominant and recessive genetic model in the common case group and severe case group

**Gene**	**SNPs**	**Dominant model (MN + NN vs MM)**		**Recessive model (NN vs MM + MN)**	
		**OR (95% CI)**	***P* value**	**OR (95% CI)**	***P* value**
*SCARB2*	**rs17001551**	0.331 (0.117–0.942)	**0.038**	–	–
*SCARB2*	rs35583533	0.987 (0.386–2.526)	0.979	–	–
*SCARB2*	rs3733256	0.344 (0.113–1.051)	0.061	–	–
*SCARB2*	rs6825004	0.865 (0.343–2.179)	0.758	–	–
*SCARB2*	rs8475	0.532 (0.190–1.489)	0.230	1.403 (0.270–7.292)	0.688
*SCARB2*	rs894251	0.672 (0.219–2.057)	0.486	0.657 (0.180–2.402)	0.525
*SCARB2*	**rs74719289**	0.297 (0.096–0.916)	**0.035**	–	–
*SCARB2*	rs76229059	1.011 (0.383–2.669)	0.982	2.172 (0.261–18.103)	0.473
*SCARB2*	rs1051326	0.552 (0.195–1.566)	0.264	0.962 (0.228–4.056)	0.958
*SCARB2*	rs3796498	0.673 (0.242–1.872)	0.449	0.615 (0.070–5.389)	0.661
*SCARB2*	rs9991821	1.043 (0.382–2.848)	0.935	–	–
*SCARB2*	rs17001640	0.727 (0.260–2.035)	0.544	1.773 (0.341–9.217)	0.496
*SCARB2*	rs6824953	0.862 (0.342–2.174)	0.753	–	–
*SCARB2*	rs894250	0.303 (0.106–0.867)	0.026	–	–
*SCARB2*	**rs3733255**	0.299 (0.097–0.921)	**0.035**	–	–
*SCARB2*	rs11547135	0.344 (0.103–1.148)	0.083	0.547 (0.140–2.130)	0.384
*SCARB2*	rs1465922	0.606 (0.232–1.584)	0.307	–	–
*SELPLG*	rs2228315	0.732 (0.276–1.940)	0.530	0.894 (0.177–4.516)	0.892
*SELPLG*	rs3782522	0.236 (0.065–0.850)	0.027	0.413 (0.079–2.150)	0.293
*SELPLG*	rs765267	0.696 (0.277–1.753)	0.442	–	–
*SELPLG*	rs8179133	0.900 (0.346–2.344)	0.830	1.363 (0.163–11.366)	0.775
*SELPLG*	rs4964269	0.404 (0.126–1.295)	0.127	0.915 (0.196–4.284)	0.911
*SELPLG*	rs7138370	0.804 (0.308–2.096)	0.655	1.119 (0.133–9.428)	0.918
*SELPLG*	rs1981758	0.731 (0.283–1.885)	0.517	1.532 (0.184–12.735)	0.693
*SELPLG*	rs8179141	0.561 (0.211–1.491)	0.246	–	–

Bold values indicate statistical significance. SNP: Single-nucleotide polymorphisms; M: Major allele; N: Minor allele; OR: Odds ratio; CI: Confidence interval; *X^2^*: Chi-square test.

For rs74719289, the alleles are A and G. The A allele is the minor allele. The genotype frequencies of the GG, GA, and AA in the severe case group were 91.01%, 8.99%, and 0.00%, while they were 75.00%, 25.00%, and 0.00% (*P* ═ 0.0266) in the common case group (Table 3). The A allele has a lower frequency in the population and is considered as a mutant gene. Patients carrying this mutation experienced milder symptoms in a dominant model (AA + GA vs GG, OR 0.297; 95% CI 0.096–0.916; *P* ═ 0.035). This difference was not statistically significant in a recessive model (AA vs GA + GG). Therefore, we suggest that if the A allele is associated with the severity of EV71 infection, it might play a role in a dominant model (Table 4).

For rs3733255, the alleles are T and C. The T allele is the minor allele. The genotype frequencies of the CC, CT, and TT in the severe case group were 90.36%, 9.64%, and 0.00%, respectively, while they were 73.68%, 23.62%, and 0.00% (*P* ═ 0.0272) in the common case group, respectively (Table 3). The T allele has a lower frequency in the population and is considered as a mutant gene. Patients carrying this mutation experienced milder symptoms in a dominant model (TT + CT vs CC, OR 0.299; 95% CI 0.097–0.921; *P* ═ 0.035). This difference was not statistically significant in a recessive model (TT vs CT + CC). Therefore, we suggest that if the T allele is associated with the severity of EV71 infection, it might play a role in a dominant model (Table 4).

No significant differences in genotype frequency for the remaining SNPs were detected between the severe case group and the common case group (Table 3).

## Discussion

Glycosylation and pH-dependent conformational changes in SCARB2 play an important role in the attachment and uncoating of EV71 [21]. EV71 infection in MAF transgenic mice expressing the human *SCARB2* gene leads to ataxia, paralysis, and death in animal experiments [6]. We studied the correlation between *SCARB2* gene polymorphisms and EV71 infection, and the results showed that the allele and genotype frequencies of rs74719289, rs3733255, and rs17001551 were significantly different between the common case group and the severe case group. Further analysis revealed that the frequency of MAF for these sites in the severe case group was significantly lower than in the common case group, and the corresponding ORs were all less than one. This indicates that *SCARB2* plays an important role in the pathogenesis of this EV71 infection, and that these polymorphism sites may play a protective role in the development of HMFD.

Expression of the human *SELPLG* gene in transgenic mice can enhance virus replication and aggravate symptoms at the early stage of mouse-adapted EV71 strain infection [22]. rs2228315 is a SNP hotspot in the study of the *SELPLG* gene polymorphism, which is close to the binding region of PSGL-1 and P-selectin [23] and related to their interaction. Eight TagSNPs of the *SELPLG* gene, including rs2228315, were selected for our study. No significant differences in allele frequency and genotype frequency were found between the common case group and the severe case group. Therefore, we conclude that the *SELPLG* gene is not closely related to the severity of HFMD.

Several studies found that after EV71 infects the human body, it first replicates in the intestinal or respiratory mucosa and then transfers to various tissues, such as the CNS, through hematological dissemination or neural pathways [24], causing degeneration, necrosis, or apoptosis of neurons [25, 26]. When the internalized receptor complex is formed, EV71 is uncoated. SCARB2 plays an important role in the binding of EV71 to the receptor, virus internalization, and uncoating [21]. In contrast, PSGL-1 functions as an attachment receptor, that supports EV-71 binding to the cell surface but does not initiate uncoating [27], and does not directly contribute to the replication or dissemination of the virus in vivo. Therefore, we believe that the severity of EV71 infection with HFMD is more closely related to the *SCARB2* gene. Notably, our research found the genetic polymorphisms in *SCARB2* (rs74719289, rs3733255, and rs17001551) that were associated with the course of HFMD were all located in 3′ untranslated regions (3′UTRs) of the genes. Research shows that 3′UTRs can play an important role in the regulation of biological complexity, such as mRNA localization and mRNA stability and translation, even by establishing 3′UTR-mediated protein–protein interactions to regulate diverse protein features [28]. We hypothesize that these SNPs might regulate the expression or function of SCARB2. The *SCARB2* gene mutation may reduce the expression level of SCARB2 protein or its binding efficiency to EV71, and it impairs the attachment and the intracellular uncoating of EV71, thereby reducing the severity of the disease. Therefore, we conclude that the *SCARB2* gene polymorphism has a protective effect on the occurrence of the disease, and further studies are needed to clarify the mechanism.

Although there were significant differences in NEUT, RBC, and HGB between the common case group and the severe case group, these three indicators fell in the range of normal references. Therefore, we believe that although these three indicators might be related to the development of HMFD, they are not the key factors in the severity of HMFD. In routine blood tests, the LYMPH and PLT increased, and RBCs showed small cell morphology (MCV and MCH decreased). This phenomenon, combined with the clinical manifestations, might have some clinical reference significance for the diagnosis of HFMD.

Yen et al. [18] studied the *SCARB2*, *SELPLG*, and Annexin A2 gene polymorphisms in HMFD patients with EV71 infection in Taiwan and found that rs6824953 and rs11097262 of the *SCARB2* gene are related to susceptibility to EV71 infection, while rs7137098 and rs8179137 of the *SELPLG* gene are related to the severity of HMFD. However, our study found that the severity of EV71 infection is related to rs74719289, rs3733255, and rs17001551 of the *SCARB2* gene but not to the *SELPLG* gene. There were some key differences between Yen’s study and our study. First, there were different diagnostic criteria. In Yen’s study, the mild group experienced uncomplicated HFMD/HA, febrile illness, or mild CNS involvement with myoclonic jerk or aseptic meningitis [18]. However, in our study, the severe cases had CNS involvement, with symptoms including listlessness, drowsiness, weak sucking, hyperarousal, headache, vomiting, etc. Thus, the two studies had different groups of subjects based on different diagnostic criteria. Additionally, Yen’s mild cases group included some of our severe cases. This is the main reason for the inconsistency between the two studies. Second, we did not set up a healthy group to study the susceptibility to EV71 infection. We believe that the occurrence of HMFD is largely determined by exposure levels to pathogenic doses of EV71. Thus, environmental factors, such as the hygiene habits of children and caregivers, are directly related to the occurrence of HMFD. Therefore, it is meaningful to discuss individual susceptibility under the premise that the possibility of viral infection is equal. Third, there were differences in the genetic backgrounds of the research cases. Yen’s cases are from Taiwan, and our cases are from Yunnan Kunming. In our cases, 86.16% were of Han nationality, and 13.84% were mainly of the Yi ethnic group (http://tjj.km.gov.cn/c/2019-09-18/3012515.shtml). Therefore, our cases differ from the ethnic composition of Taiwan. The different genetic backgrounds of the study cases can lead to differences in the gene polymorphism itself, ultimately producing different results. In summary, the two studies chose to examine *SCARB2* and *SELPLG* genes for TagSNPs and studied their correlation with EV71 infection in HMFD. However, due to differences in categorizing and different genetic backgrounds of the study cases, the study results are inconsistent. This reminds us that unified clinical diagnostic criteria are the premise for comparing the results of different studies. In addition to the *SCARB2* and *SELPLG* genes, EV71 infection may be related to other major genes.

## Conclusion

Briefly, we conclude that the rs74719289, rs3733255, and rs17001551 polymorphisms of the *SCARB2* gene are related to the development of EV71 infection and that mutation of the *SCARB2* gene can play a protective role by inhibiting the development of EV71 infections in HMFD. As the pathogenesis of EV71 infection of HMFD is very complicated, future studies would benefit from expanding the sample size, unifying diagnostic criteria, adding the inapparent infection group, and conducting more research to further clarify the factors influencing HMFD.

## Supplemental Data

**Table S1:** SNPs information

**SNP site**	**Gene**	**Allele**	**Region**	**Amino acid changes**
**rs17001551**	*SCARB2*	A/G	3’UTR	
**rs35583533**	*SCARB2*	C/T	Intron	
**rs3733256**	*SCARB2*	C/G	3’UTR	
**rs6825004**	*SCARB2*	G/C	Intron	
**rs8475**	*SCARB2*	A/T	3’UTR	
**rs894251**	*SCARB2*	A/G	Intron	
**rs74719289**	*SCARB2*	A/G	3’UTR	
**rs76229059**	*SCARB2*	A/G	Intron	
**rs1051326**	*SCARB2*	C/G	3’UTR	
**rs3796498**	*SCARB2*	T/C	Intron	
**rs9991821**	*SCARB2*	A/G	Intron	
**rs17001640**	*SCARB2*	A/G	Intron	
**rs6824953**	*SCARB2*	C/G	Intron	
**rs894250**	*SCARB2*	C/A	Intron	
**rs3733255**	*SCARB2*	C/T	3’UTR	
**rs11547135**	*SCARB2*	C/T	5’UTR	
**rs1465922**	*SCARB2*	A/G	3’UTR	
**rs2228315**	*SELPLG*	T/C	Extron	Met62Ile
**rs3782522**	*SELPLG*	T/C	Intron	
**rs765267**	*SELPLG*	A/G	3’UTR	
**rs8179133**	*SELPLG*	A/G	Intron	
**rs4964269**	*SELPLG*	A/G	Intron	
**rs7138370**	*SELPLG*	C/G	Intron	
**rs1981758**	*SELPLG*	T/C	Intron	
**rs8179141**	*SELPLG*	T/C	Intron	

The allele is described as minor allele/major allele; SNPs: Single-nucleotide polymorphisms; UTR: Untranslated regions.

**Table S2:** SNPs primers

**Sites**	**Forward**	**Reverse**	**Polymorphism**	**Direction**	**Product**	**Primer of extension**
**rs3733255**	CTTTAACCTCTGGCCAGAATG	TTCTGTGTTTCAGGGAACAGC	[C/T]	F	CT	TTTTTTTTTTTGTTCCTATCACTTGCCAGCGC
**rs3733256**	CTTTAACCTCTGGCCAGAATG	TTCTGTGTTTCAGGGAACAGC	[C/G]	F	CG	TTTTTTTTTTTTTTTTTTTTACAAGCCTGCAAGGAGGTGGAG
**rs4964269**	ACTTAGCGGCTGTGTAAACTC	TCCATTTCCTCTGCTCATCTG	[A/G]	F	AG	TTTTTTTTTTCCAGCCGGGGGTACTTTATCTG
**rs8475**	GAACCTTTAGATACTCCAACT	AGCCTGGCGACAGAGTGAGA	[A/T]	F	AT	TTTTTTTTTTCTAGATAATTGGGCATGTCTTA
**rs74719289**	TTGTCACAGGAAGTATAGGGC	GGAAATCCATCTATCTACAGCC	[A/G]	F	AG	CTCTATTCAGTGAGTGACAGTGA
**rs1051326**	TTGTCACAGGAAGTATAGGGC	GGAAATCCATCTATCTACAGCC	[C/G]	F	CG	TTTTTTTTTTCTGAGGAAGGAACTTGTAAAAA
**rs17001551**	GAAGACTGAGTTTTCCTGGAAG	CATTCACCGAACTTTGTGCTC	[A/G]	F	AG	TTTTTTTTTTTTTTTTTTTTCTACCATTTTACCCTGGGTCCC
**rs35583533**	GCCATGATGATGTAGGGTATG	TTGGTTTGCTAACAGGAGGAC	[C/T]	F	CT	GACTAAACATCCAGTGTGTAAC
**rs894251**	CTCACCTCTCATGCTACATTG	ACTTTCCACTTTCGGTTGTCC	[G/A]	R	CT	AGAAATCAAAGGGCAAGAACCA
**rs894250**	CTCACCTCTCATGCTACATTG	ACTTTCCACTTTCGGTTGTCC	[C/A]	R	GT	TTTTTTTTTTACTCAAACTAGCTCTGGCAAGA
**rs6824953**	TAGGTGGGTGCAAAGTAACTG	TTCCCAATGTACTGGAAGCTC	[C/G]	F	CG	TTTTTTTTTTTTTTTTTTTTTAAATAGTAGACAGTCAGAGAC
**rs8179141**	AGGTTGCAGTGAGCTGAGATAG	AATTGATTTGCCTCCCTCTCC	[T/C]	R	AG	TCTTTCTACACCCCAGTGGATT
**rs3796498**	ACTATGCAGTGTAAGCAGTGG	GGGTCTTAGGCACTTGAAAAG	[T/C]	F	CT	ACAGATACTTTATTGGAATACA
**rs17001640**	CCTCAGTAGTGGCAAAATAGC	TGTTCACTACACACAGCTCAG	[A/G]	R	CT	TTTTTTTTTTTGTGTACCCAAATGGAAGCTTA
**rs76229059**	AGATGGGAAAAGTGGGTTCAG	TCTCCTGAGTTGCCTCTATTC	[A/G]	F	AG	TTTTTTTTTTTCAAGAAAAATCTCAGACTAAG
**rs9991821**	AGATGGGAAAAGTGGGTTCAG	TCTCCTGAGTTGCCTCTATTC	[A/G]	F	AG	TTTTTTTTTTTTTTTTTTTTACAGATTTAAGATATCTTTACA
**rs8179133**	AGAATCACTTGAGCCTAGGAG	CAAAGTGCTGGGATTACAGGG	[G/A]	R	CT	TGAAACCAACATAGGCCTGGCA
**rs7138370**	TATGAGCACACTCACAGCTAC	CCGTGTTTCTGTGTTATTCTCC	[C/G]	F	CG	TTTTTTTTTTGTCAGGGCCGGACTTGCAGCAG
**rs2228315**	ATGATTTCCTGCCAGAAACGG	ATCTCCATAGCTGCTGAATCC	[T/C]	F	CT	TGGTGTCAGTGCTGTTCCTCAG
**rs3782522**	TCTGCAGAGGCATTTAGTGAG	AAACTTCCAGAAGGCAGGAAC	[T/C]	R	AG	AGTGGTTGCCAGCCACTGGGGC
**rs1981758**	TCCTCCACACAGCCTAGATG	CCTTCCACCTCTCCTCTTCC	[T/C]	F	CT	TTTTTTTTTTGGCCCAGGCCCCATCTAGACCC
**rs765267**	AAGTTCAGCAAAGGAAGCCG	AAACCTCAGAGAGCGGAAGG	[A/G]	F	AG	TTTTTTTTTTCCACTCTGGGCCCAGCCTAGCA
**rs11547135**	AAGGAAACCGAAACCGAGTC	TACTCTGGTCTACAGCCTTC	[T/C]	R	AG	GGCGTGGCGCCGAAGGGTCCCG
**rs1465922**	CAACTGCAAGGAGGGAGGAG	CGAAGGAAACCGAAACCGAG	[A/G]	F	AG	TTTTTTTTTTGCTCCGCGGCCTGGCGAGCGCG
**rs6825004**	TACATATTCCACAAATATTCA	CTCAGTCTCAGGTATGTCCT	[C/G]	F	CG	TTTTTTTTTCCAGTACATTGGGAAGAAAGA

SNPs: Single-nucleotide polymorphisms.
